# Supplementary material for: Evaluation of the replicability of systematic reviews with meta-analyses of the effects of health interventions
Source: Res Synth Methods. 2026 Jan 9;17(3):538–56. doi: 10.1017/rsm.2025.10064 (PMC13126212; doi:10.1017/rsm.2025.10064)
Supplement: Hamilton et al. supplementary material [file S1759287925100641sup001.pdf]

SUPPLEMENTARY INFORMATION

Appendix to: Hamilton DG, McKenzie J, Nguyen PY, Rethlefsen ML, McDonald S, Brennan SE, Fidler FM, Higgins JPT, Kanukula R, Karunananthan S, Maxwell LJ, Moher D, Nakagawa S, Nunan D, Tugwell P, Welch VA, Page MJ. Evaluation of the replicability of systematic reviews with meta-analyses of the effects of health interventions. 2025.

TABLE OF CONTENTS

SUPPLEMENTARY TABLES *3*

Supplementary Table 1. Deviations from the planned methodology. 3

Supplementary Table 2. Results of the literature search replications. 8

Supplementary Table 3. Investigations of the 13 different screening decisions and the suspected reasons for their cause. 10

Supplementary Table 4. Investigations of the reasons for the data extraction discrepancies. 13

Supplementary Table 5. Percentage differences between the original review's and replicators' summary estimate and confidence interval widths. 18

Supplementary Table 6. Discordance between P values for the test for overall effect from the original review's and Replicator 1's meta-analyses. 20

Supplementary Table 7. Factors associated with the eight blinded replicated meta-analyses classified as not fully replicable. 22

Supplementary Table 8. Factors associated with the five unblinded replicated meta-analyses classified as not fully replicable. 23

SUPPLEMENTARY FIGURES *24*

Supplementary Figure 1. Scatter plot of the number of records retrieved by the original reviewers (square symbols) and the first (plus signs) and second (cross signs) replicators by database. 24

Supplementary Figure 2. Results of the original and replicated index meta-analyses for the review by AlAnouti et al. (2020). 25

Supplementary Figure 3. Results of the original and replicated index meta-analyses for the review by Dohos et al (2021). 26

Supplementary Figure 4. Results of the original and replicated index meta-analyses for the review by Gingold-Belfer et al. (2021). 27

Supplementary Figure 5. Results of the original and replicated index meta-analyses for the review by Goldberg et al. (2020). 28

Supplementary Figure 6. Results of the original and replicated index meta-analyses for the review by Ioannou et al. (2021). 29

Supplementary Figure 7. Results of the original and replicated index meta-analyses for the review by Jakubczuk et al. (2020). 30

Supplementary Figure 8. Results of the original and replicated index meta-analyses for the review by Minozzi et al. (2020). 31

Supplementary Figure 9. Results of the original and replicated index meta-analyses for the review by Wang et al. (2020). 32

Supplementary Figure 10. Results of the original and replicated index meta-analyses for the review by Yekeduz et al. (2020). 33

Supplementary Figure 11. Results of the original and replicated index meta-analyses for the review by Zhou et al. (2021). 34

Supplementary Figure 12. Results of the original and sensitivity meta-analyses for the review by AlAnouti et al. (2020). 35

Supplementary Figure 13. Results of the original and sensitivity meta-analyses for the review by Wang et al. (2020). 36

SUPPLEMENTARY TABLES

Supplementary Table 1. Deviations from the planned methodology.

Section	Original methods	Revised methods	Reason for modification
Sampling frame	We will restrict the sampling frame to systematic reviews that included 5-10 studies in the index meta-analysis.	We restricted the sampling frame to systematic reviews that included 2-10 studies in the index meta-analysis.	Increasing the range of studies that could be included in the index meta-analyses gave us a larger sample to draw from (227 of the 300 systematic reviews in REPRISE Study 1 had between 2 and 10 studies in the index meta-analysis, whereas 130 systematic reviews had between 5 and 10 studies in the index meta-analysis). Furthermore, restricting eligibility to systematic reviews with 5-10 studies in the index meta-analysis would have limited the generalisability of the sample.
Sampling frame	We will include systematic reviews in the sampling frame regardless of how completely the search methods and results were reported.	We restricted the sampling frame to systematic reviews in which authors reported the full Boolean search strategy for each database searched and the number of results yielded by each database (or a combination of them).	We had originally planned for replicators to attempt to construct search strategies from incompletely reported information (e.g. list of keywords only). We subsequently decided that it was more valuable to determine whether completely reported search strategies could be replicated, because if not, chances of replication success for incompletely reported ones would likely be even lower. Furthermore, for systematic reviews in which authors did not report the number of records yielded per database, we would be unable to compare the number of records yielded originally and upon replication.
Sampling frame	We will draw a random sample of 32 systematic reviews for replication.	We planned to draw a random sample of 10 systematic reviews for replication.	Doing so was subsequently considered more feasible. Unexpectedly, only 10 systematic reviews met the

Section	Original methods	Revised methods	Reason for modification
			eligibility criteria for inclusion, so we did not need to draw a random sample.
Crowdsourcing of reviewers	We planned to recruit 60 replicators via various avenues, with each team including at least 2 members. Each team would conduct 2 replications each.	We recruited 2 information specialists and 2 systematic reviewers who had previously worked closely with the study coordinator to conduct all 10 replications.	Doing so gave the study coordinator confidence that the replicators had the necessary expertise to carry out all tasks.
Data collection	We will share all instructions and PDF copies of full-text reports with replicators via the Open Science Framework repository.	Instructions were shared via Google Drive instead.	For convenience, as all replicators routinely use Google Drive in their day-to-day work.
Data collection	One of the REPRISE investigators (the study coordinator) will assemble all the information and files necessary for the replication of the 32 systematic reviews.	After completing all replications blinded to the results of the original review ("blinded replications"), one of the replicators checked the information extracted by the study coordinator for accuracy.	Doing so ensured that replications conducted after results of the original review were known ("unblinded replications") were guided by accurate information.
Data collection	We will also invite the authors of the original systematic reviews to provide us with a file containing all their screening decisions, if not already made publicly accessible.	Authors of the original systematic reviews were not contacted to provide such information.	We planned to use this information from the original reviewers to compare their title/abstract screening decisions with the replicators' decisions. However, we subsequently made the decision to not attempt to replicate the title/abstract screening phase of the systematic reviews, due to the substantial time investment required to do so.
Replication methods	Of the 32 reviews included, 30 will be replicated by one team each (with detailed information provided about the methods). The remaining two reviews will each be replicated by 15 teams (with	We decided not to perform the Many Analysts replications.	Not doing the Many Analysts replications would save time and resources.

Section	Original methods	Revised methods	Reason for modification
	minimal information provided about the methods) in a Many-Analysts style.		
Replication methods	If full search strategies are not reported, replicators will be permitted to request the strategies from the authors of the original systematic review (with the contact made via one of the REPRISE investigators).	Replicators of the search will not contact authors of the original systematic review for non-reported search strategies.	There was no need to contact the original reviewers for the non-reported search strategies, because systematic reviews were only included if all search strategies were fully reported.
Replication methods	Replicators will document…any errors detected when rerunning each original search strategy….	Replicators were not prompted via the replicator form given to them to record any errors detected when rerunning each original search strategy.	This was accidentally omitted from the replicator form by the study coordinator. However, the search replicators did end up recording errors they encountered on the form.
Replication methods	Replicators will document…the number of unique citations after duplicates were removed.	Replicators will not document the number of unique citations after duplicates were removed.	To obtain such information would have required the search replicators to download the number of citations yielded by each database and then remove duplicates via software. Doing so would have taken a long time in many cases (e.g. for databases where citations can only be exported in batches of 1000, and the total number of citations yielded is 15000), so we removed this requirement to save the search replicators' time.
Replication methods	Two team members will screen independently a random sample of a maximum of 100 titles and abstracts yielded from the searches against the inclusion criteria reported in the original review, and record their screening decisions ('include', 'exclude', or 'unsure').	No screening of titles and abstracts yielded from the searches occurred.	We subsequently decided that replicating the screening of 100 titles and abstracts was a waste of time. We assumed that in many cases, all the titles and abstracts screened would be ineligible, which would have prevented us from people able to use agreement statistics (e.g. Kappa) to determine replication success.

Section	Original methods	Revised methods	Reason for modification
Replication methods	‘Results fully replicable’ was originally defined as “no difference [with allowance for trivial discrepancies such as those due to computational algorithms] is observed between the original and recalculated meta-analytic effect estimate, its 95% confidence interval and inferences about heterogeneity reported in the original review);	Meta-analyses where both the original and replicated summary estimate of the and its 95% confidence interval width differed by 10% or less were classified as “results fully replicable”.	We subsequently decided to use a cutoff value to remove subjectivity in the judgements.
Replication methods	A REPRISE investigator and the team will also independently specify whether they believe the observed difference between the original and recalculated summary estimate and its precision was meaningful, that is, would lead to a change in the interpretation of the results (classified as ‘difference meaningful’ or ‘difference not meaningful’).	A difference between the original and replicated summary estimate was considered “meaningful” if either the statistical significance or direction differed.	We subsequently decided to use objective criteria to remove subjectivity in the judgements.
Interview methods	Once teams complete both replications, investigators will conduct semi-structured interviews (of approximately 30 min duration) with replicators to discuss the analytical steps they took and to understand the decision-making processes used when synthesising the data.	No interviews were conducted.	The interviews were designed mainly to capture replicators thoughts about the findings of the Many Analysts replications. Given these replications were not done, the interviews were considered less of a priority.
Data analysis	We will assess agreement between the original and replicated review in the number of citations yielded from each database, in total and once duplicates were removed, by calculating the weighted Kappa statistic and percentage agreement (both metrics will be presented with 95% confidence intervals).	We determined agreement in search results by calculated the percentage difference between the original reviewer’s and replicators’ search results. Percentage differences less than or equal to 10% were considered a successful replication.	Our plan to calculate a weighted Kappa statistic for search results was written in error. We used a 10% cutoff for comparability with a similar study (PMID: 38052277).

Section	Original methods	Revised methods	Reason for modification
Data analysis	We will assess agreement between the original and replicated review in screening decisions (where available) for the subset of titles and abstracts and full-text reports screened by replicators by calculating the weighted Kappa statistic and percentage agreement (both metrics will be presented with 95% confidence intervals).	We calculated an unweighted Kappa statistic and percentage agreement to assess agreement between the original and replicated screening decision for full text reports.	Titles and abstracts were not screened by replicators. We calculated unweighted Kappa because there were only two response options for screening: "Include" or "Exclude".
Data analysis	We will calculate the frequency and percentage (with 95% confidence intervals) of …, (ii) search strategies replicators needed to reconstruct or adapt based on the partial information available	We did not calculate the frequency of search strategies replicators needed to reconstruct or adapt based on the partial information available.	None of the search strategies replicated needed to be reconstructed or adapted based on partial information available, as all were completely reported.
Data analysis	We will calculate agreement between the original and replicated meta-analytic effects, displayed using Bland-Altman plots.	We did not calculate agreement between the original and replicated summary estimates using Bland-Altman plots.	We subsequently considered Bland-Altman plots would no longer be useful to present given the smaller number of systematic reviews included (n=10) and the variation in effect measures used.

Supplementary Table 2. Results of the literature search replications.

Review author	Database	Number of records retrieved			Percentage difference			Speculated cause of discrepancy	
		Original reviewers	Replicator 1	Replicator 2	Reviewers & Replicator 1	Reviewers & Replicator 2	Replicator 1 & Replicator 2	Replicator 1	Replicator 2
Dohos	PubMed	8889	2921	2927	-67%	-67%	0%	Reviewer error	Reviewer error
	Embase	11907	5722	12722	-52%	7%	122%	Different field tags	No discrepancies
	CENTRAL	1778	949	942	-47%	-47%	-1%	Reviewer error	Reviewer error
	Web of Science	9538	3207	2803	-66%	-71%	-13%	Reviewer error	Reviewer error
	Scopus	14598	3673	3673	-75%	-75%	0%	Reviewer error	Reviewer error
Ioannou	PubMed	912	908	910	0%	0%	0%	No discrepancies	No discrepancies
	Embase	1282	1281	1320	0%	3%	3%	No discrepancies	No discrepancies
	CINAHL	216	169	178	-22%	-18%	5%	Default settings	Default settings
	PsycINFO	1111	1097	973	-1%	-12%	-11%	No discrepancies	Different platform
	CENTRAL	248	278	241	12%	-3%	-13%	Different start date	No discrepancies
Yekeduz	MEDLINE	3412	3940	3977	15%	17%	1%	Unreported limit	Unreported limit
Zhou	CINAHL	29	24	22	-17%	-24%	-8%	Default settings	Default settings
	CENTRAL	155	141	157	-9%	1%	11%	No discrepancies	No discrepancies
	Embase	56	69	70	23%	25%	1%	Unknown	Unknown
	ProQuest	10	71	32	610%	220%	-55%	Different resources searched	Different resources searched
	PubMed	55	36	36	-35%	-35%	0%	Platform update	Platform update
	Scopus	137	101	101	-26%	-26%	0%	Unknown	Unknown
	Web of Science	41	2209	100	5288%	144%	-95%	Incomplete syntax	Incomplete syntax
Minozzi	CDAG Register	5	NA	NA	NA	NA	NA	NA	NA
	CENTRAL	87	245	243	182%	179%	-1%	Unknown	Unknown
	MEDLINE	74	75	75	1%	1%	0%	No discrepancies	No discrepancies
	Embase	97	96	90	-1%	-7%	-6%	No discrepancies	No discrepancies
	CINAHL	248	317	257	28%	4%	-19%	Different sources	No discrepancies

	PsycINFO	34	37	37	9%	9%	0%	No discrepancies	No discrepancies
	Web of Science	88	88	91	0%	3%	3%	No discrepancies	No discrepancies
Wang	PubMed	1980	2114	2116	7%	7%	0%	No discrepancies	No discrepancies
	Embase	1408	9759	1311	593%	-7%	-87%	Did not fix errors	No discrepancies
	CENTRAL	42	87	74	107%	76%	-15%	Unknown	Unknown
AlAnouti	MEDLINE	854	841	841	-2%	-2%	0%	No discrepancies	No discrepancies
	PubMed	1028	991	991	-4%	-4%	0%	No discrepancies	No discrepancies
	CINAHL	852	755	785	-11%	-8%	4%	Unknown	No discrepancies
	Embase	941	909	918	-3%	-2%	1%	No discrepancies	No discrepancies
	CENTRAL	900	857	951	-5%	6%	11%	No discrepancies	No discrepancies
Goldberg	PubMed, CINAHL & PsycINFO	5389	6910	4852	28%	-10%	-30%	Unknown	Unknown
	PubMed	NA	3130	2965	NA	NA	-5%	NA	NA
	CINAHL	NA	743	506	NA	NA	-32%	NA	NA
	PsycINFO	NA	3037	1381	NA	NA	-55%	NA	NA
	Web of Science	2167	2334	2620	8%	21%	12%	No discrepancies	Different resources searched
	Scopus	6650	5056	5046	-24%	-24%	0%	Different field tags	Different field tags
	CENTRAL	380	390	373	3%	-2%	-4%	No discrepancies	No discrepancies
Jakubczyk	PubMed	473	634	635	34%	34%	0%	Undisclosed limits	Undisclosed limits
	Embase	286	341	335	19%	17%	-2%	Undisclosed limits	Undisclosed limits
	PubMed	98	98	98	0%	0%	0%	No discrepancies	No discrepancies
Gingold-Belfer	MEDLINE	96	98	98	2%	2%	0%	No discrepancies	No discrepancies
	Embase	453	434	455	-4%	0%	5%	No discrepancies	No discrepancies
	Web of Science	61	102	98	67%	61%	-4%	Different resources searched	Different resources searched
	CENTRAL	50	26	24	-48%	-52%	-8%	Unknown	Unknown

Supplementary Table 3. Investigations of the 13 different screening decisions and the suspected reasons for their cause.

Review	Study	Original reviewers	Blinded replicators	Discrepancy reason	Discrepancy details	Unblinded replicators	Changed decision
AlAnouti 2020	Yin 2016	Exclude	Include	Coordinator error	The REPRISE study coordinator missed guidance in the review's results section which outlined that they excluded this study due to the follow up period being too long (i.e., the eligible time frames for the outcome was not specified in the methods section).	Exclude	Yes
Dohos 2020	Candy 1995	Include	Exclude	Replicator error	Both replicators missed that the study authors did report the outcome of interest. That is, how many participants responded to the 12-weeks prednisolone treatment, and of them, the number of people who stayed in remission after receiving azathioprine or placebo.	Include	Yes
Dohos 2020	O'Donoghue 1978	Include	Exclude	Replicator error	This study included a group which received both an immunomodulator and anti-inflammatory drugs. The original reviewers did not provide specific guidance as to whether anti-inflammatory drugs are an acceptable co-intervention for IM monotherapy. Given the lack of guidance and content expertise, the blinded replicators decided to exclude this study. After reading the review report and other relevant literature, we decided that this was likely considered an acceptable co-intervention.	Include	Yes
Ioannou 2020	Elsenga 1982	Include	Exclude	Incomplete eligibility criteria	The original reviewers explicitly stated that studies would only be included if they followed DSM criteria. It was not specified in the primary study whether study participants had depression as defined according to DSM criteria. Consequently, the replicators decided to exclude this study due to a lack of information (note that an attempt was made to contact the study authors which was unsuccessful). When the original reviewers were asked about the eligibility assessment of this study, they stated via email that they decided to include the study based on information contained in the study author's dissertation. A clinical psychologist was also consulted to evaluate the similarity of the diagnostic criteria used by the study authors in comparison to the DSM. Their view was also that the two criteria (Elsenga's and DSM III) were sufficiently similar.	Include	Yes
Ioannou 2020	Kragh 2017a	Include	Exclude	Unclear eligibility criteria	The original reviewers explicitly stated that studies would only be included if they used DSM criteria to diagnose depression. The study authors stated that depression was diagnosed according to ICD-10 criteria. Therefore, the blinded replicators excluded this study. After unblinding, the replicators	Exclude	No

Review	Study	Original reviewers	Blinded replicators	Discrepancy reason	Discrepancy details	Unblinded replicators	Changed decision
					noted that this study was included in the original review. Following this discovery the replicators looked at the two diagnostic criteria, as well as consulted with a clinical psychologist, and maintained their view that ICD-10 and DSM III criteria were too dissimilar (as well as at direct odds with the instructions given) and so did not include this study in the unblinded replication either. Consequently, it appears that either the original reviewers did not follow their own eligibility criteria, or the allowance of the ICD-10 criteria was an undeclared change to the eligibility criteria.		
Ioannou 2020	Wu 2009	Include	Exclude	Incomplete eligibility criteria	The intervention in this study consisted of sleep deprivation and sleep phase advance. While the original reviewers stated that light therapy was considered an acceptable co-intervention, they did not provide guidance on the acceptability of other chronotherapies (e.g., sleep phase advance, sleep time stabilisation). Given the lack of information, the blinded replicators decided to exclude this study. After being unblinded and reading the limitations section in the discussion section of the review report it appears that the original reviewers also considered sleep phase advance (as well as sleep time stabilisation) to be an acceptable chronotherapeutic co-intervention in addition to 'light therapy'.	Include	Yes
Wang 2020	Akiyama 1994	Exclude	Include	Coordinator error	The REPRISE study coordinator failed to notice that the original reviewers indicated that they would exclude studies with a Newcastle-Ottawa Score less than six from the meta-analysis.	Exclude	Yes
Wang 2020	Fujita 2003	Exclude	Include	Coordinator error	The REPRISE study coordinator failed to notice that the original reviewers indicated that they would exclude studies with a Newcastle-Ottawa Score less than six from the meta-analysis.	Exclude	Yes
Wang 2020	Koterazawa 2019	Exclude	Include	Coordinator error	The REPRISE study coordinator failed to notice that the original reviewers indicated that they would exclude studies with a Newcastle-Ottawa Score less than six from the meta-analysis.	Exclude	Yes
Yekeduz 2020	Lee 2020b	Exclude	Include	Replicator error	The replicators failed to notice that this study’s population overlapped with the population from the Lee 2020a report (which was also included in the original review). The replicators assume Lee 2020a was included in place of this study as it reports on a bigger sample size and a broader cohort (i.e., not just haematological malignancies).	Exclude	Yes

Review	Study	Original reviewers	Blinded replicators	Discrepancy reason	Discrepancy details	Unblinded replicators	Changed decision
Zhou 2021	Hoseini 2016	Exclude	Include	Unclear	The original reviewers state in the narrative synthesis section (3.8) that they were unable to retrieve sufficient information for inclusion of this study. However, to the replicators, all required information appears to have been reported in the study's abstract. Therefore, it is unclear to us exactly why this study was not included by the original reviewers.	Include	No
Zhou 2021	Minatel 2009	Include	Exclude	Ambiguous eligibility criteria	The REPRISE study coordinator advised the blinded replicators that participants with "lower limb diabetic foot ulcers" were eligible for the index meta-analysis as this was stated in the eligibility criteria section in the methods section. The replicators also noted that no explicit guidance was provided regarding whether the original reviewers were interested in diabetic (neuropathic) ulcers and/or other ulcers (e.g., ulcers due to venous or arterial insufficiency) in diabetic populations. Given the presence of the term 'diabetic foot ulcer' in the criterion, the blinded replicators decided to interpret "lower limb diabetic foot ulcers" as neuropathic ulcers occurring on the foot (i.e., ulcers distal to the ankle), not leg, and hence excluded this study as it included participants with both mixed ulcers, as well as ulcers above the ankle. Upon reading the original review's supplementary material after unblinding, we noted that the original reviewers stated that "lower *limb* wounds/ulcers" were deemed eligible. After seeing this phrasing, we reasoned that the original reviewers likely intended that the phrase "lower limb diabetic foot ulcers" refer broadly to diabetic ulcers, *as well as* mixed (e.g., venous and arterial) ulcers in people with Type II diabetes, occurring anywhere on the lower extremity. However, we were not able to confirm this updated interpretation of the eligibility criteria with the original reviewers.	Include	Yes
Zhou 2021	Zhang 2013	Include	Exclude	Unclear eligibility criteria	The original reviewers explicitly stated in the detailed eligibility criteria specified in the supplement that articles in languages other than English were ineligible. As this study is written in Chinese, it was excluded by the replicators in both phases. Consequently, it appears that either the original reviewers did not follow their own eligibility criteria, or that the inclusion of foreign-language articles was an undeclared change to the eligibility criteria. We did not change our decision as both the PROSPERO entry and report say that they did not include non-English language studies.	Exclude	No

Supplementary Table 4. Investigations of the reasons for the data extraction discrepancies.

Review	Study	EE type	Original reviewers		Blinded replicators		Unblinded replicators		Discrepancy reason	Discrepancy details
			EE	95% CI	EE	95% CI	EE	95% CI		
AlAnouti 2020	Wongwiwatthananukit 2013	MD	2.04	(-31.00, 35.08)	2.04	(-29.88, 33.96)	NA	NA	Reviewer error	The original reviewers appear to have not noticed that the study authors imputed data for the 2 people in each group who withdrew prior to the Week 8 mark.
Dohos 2020	Hawthorne 1992	Risk ratio	1.39	(0.85, 2.26)	1.62	(0.95, 2.75)	1.52	(0.90, 2.57)	Incomplete data selection methods	No guidance was provided in the review report on which timepoint to choose. The blinded replicators chose the result reported in text which represents the relapse rate at eight months (i.e., the time prior to the first person dropping out). Whereas, after being unblinded, and in consultation with a biostatistician, the replicators and decided to use the data at 12 months which required removing the two censored patients. Differences between both replicators' results and the original reviewers' results likely relate to differences in the assumption about what has happened to the two people who dropped out. We assumed they didn't relapse. The review authors may not have assumed this.
Dohos 2020	O'Donoghue 1978	Risk ratio	2.44	(0.90, 6.67)	NA	NA	7.56	(1.04, 54.91)	Incomplete data selection methods	No guidance was provided on how to account for patients who dropped out. Differences between the replicators and original reviewers likely relate to the replicators assuming the five participants who exited the trial for 'reasons other than relapse' didn't relapse. The review authors may not have assumed this.
Dohos 2020	Vilien 2004	Risk ratio	2.49	(0.82, 7.55)	3.47	(0.89, 13.51)	NA	NA	Incomplete data selection methods	No guidance was provided on how to account for patients who dropped out. Differences between the replicators and original reviewers likely relate to the replicators assuming that the person who exited the trial didn't relapse. The review authors may not have assumed this.
Gingold-Belfer 2020	Perri 2001	Odds ratio	1.64	(0.85, 3.17)	1.64	(0.75, 3.62)	NA	NA	Reviewer error	It appears that the original reviewers have double counted the control group (60/90) when calculating the odds ratio.

Review	Study	EE type	Original reviewers		Blinded replicators		Unblinded replicators		Discrepancy reason	Discrepancy details
			EE	95% CI	EE	95% CI	EE	95% CI		
Goldberg 2020	Gasser 2014	SMD	2.22	(0.91, 3.53)	0.09	(-1.24, 1.42)	NA	NA	Incomplete data selection methods	No specific guidance was provided on which outcome measure to choose when multiple measures were available. Therefore, it is very likely that differences occurred due to the choice of outcome used to serve as the surrogate for 'targeted symptoms'. The replicators decided to choose the outcome scale they felt was most applicable to the population's condition of interest (e.g., depression-only scale for populations with depression, combined depression and anxiety scale for populations with both anxiety and depression) as well as to use results from the same scale from each study when multiple scales were reported. The original reviewers stated upon request for clarification that they no longer have the data for the review, so we are unable to confirm that this was the cause of the discrepancy.
Goldberg 2020	Griffiths 2016	SMD	0.86	(0.24, 1.48)	0.55	(-0.01, 1.11)	NA	NA	Incomplete data selection methods	As per Gasser 2014 comment.
Goldberg 2020	Grob 2011	SMD	0.94	(-0.06, 1.94)	1.21	(-0.04, 2.46)	NA	NA	Incomplete data selection methods	As per Gasser 2014 comment.
Goldberg 2020	Palhano-Fontes 2019	SMD	1.20	(0.52, 1.88)	1.77	(0.90, 2.64)	NA	NA	Incomplete data selection methods	As per Gasser 2014 comment.
Goldberg 2020	Ross 2016	SMD	0.98	(0.27, 1.69)	0.61	(-0.14, 1.35)	NA	NA	Incomplete data selection methods	As per Gasser 2014 comment.
Ioannou 2020	Benedetti 1997	SMD	-0.51	(-1.78, 0.77)	-0.61	(-1.90, 0.67)	NA	NA	Reviewer error	It appears that the original reviewers incorrectly extracted the Day 14 results rather than the Day 7 results. The review report specifies that the outcome of interest is the depression scores within one week of starting treatment. Study authors state that participants commenced sleep deprivation on Day 6.

Review	Study	EE type	Original reviewers EE	Original reviewers 95% CI	Blinded replicators EE	Blinded replicators 95% CI	Unblinded replicators EE	Unblinded replicators 95% CI	Discrepancy reason	Discrepancy details
Ioannou 2020	Kundermann 2008	SMD	-0.17	(-1.07, 0.73)	-0.17	(-1.08, 0.73)	-0.17	(-1.08, 0.73)	Data extraction from plot	The difference is due to a small variation in the value of the standard deviation of the control group that was estimated from Figure 2.
Wang 2020	Fujita 1995	HR	1.13	(0.67, 1.92)	1.19	(0.77, 1.83)	1.19	(0.77, 1.83)	Data extraction from plot	The difference is due to a combination of the manual extraction of data from reported Kaplan-Meier (KM) curves and the estimation of the hazard ratio and standard error from the data via Guyot's algorithm. We noted during calibration testing that extracting more datapoints from KM curves, and the addition of other pertinent data into the algorithm (e.g., data from risk tables), resulted in more accurate estimates.
Wang 2020	Igaki 2004		1.29	(0.77, 2.15)	1.25	(0.80, 1.96)	1.25	(0.80, 1.96)	Data extraction from plot	As per Fujita 1995 comment.
Wang 2020	Li 2012		0.88	(0.63, 1.23)	0.88	(0.62, 1.26)	0.88	(0.62, 1.26)	Data extraction from plot	As per Fujita 1995 comment.
Wang 2020	Shim 2010		1.35	(0.71, 2.58)	1.15	(0.64, 2.07)	1.15	(0.64, 2.07)	Data extraction from plot	As per Fujita 1995 comment.
Wang 2020	Tabira 1999		1.20	(0.65, 2.20)	1.52	(0.89, 2.57)	1.52	(0.89, 2.57)	Data extraction from plot	As per Fujita 1995 comment.
Wang 2020	Zhang 2008		1.31	(0.82, 2.10)	1.30	(0.86, 1.96)	1.30	(0.86, 1.96)	Data extraction from plot	As per Fujita 1995 comment.
Zhou 2021	Naidu 2005	SMD	4.17	(2.45, 5.89)	2.92	(1.40, 4.43)	NA	NA	Unclear	No specific guidance was provided on which timepoint to choose when outcome data from multiple timepoints were available. Consequently, the blinded replicators chose the Week 2 result to ensure consistency with other included studies (which all provided outcome data at Week 2). After this discrepancy was discovered following unblinding, Hedge's g

Review	Study	EE type	Original reviewers		Blinded replicators		Unblinded replicators		Discrepancy reason	Discrepancy details
			EE	95% CI	EE	95% CI	EE	95% CI		
										was calculated for all available timepoints to determine if a different timepoint was chosen by the original reviewers. However, we were unable to reconstruct the reported value from the review. The replicators tried to confirm with the original reviewers but received no response.

Supplementary Table 5. Percentage differences between the original review's and replicators' summary estimate and confidence interval widths. Note: Ratio measures were log-transformed (natural log) prior to calculation of the percentage difference.

				Percentage difference	
**Review**	**Analysis**	**Summary estimate**	**95% CI**	**Summary estimate**	**CI width**
Dohos 2020	Original (report)	1.85	(1.44, 2.38)	NA	NA
	Replicator 1 (blinded)	1.844	(1.336, 2.545)	-1%	28%
	Replicator 2 (blinded)	1.844	(1.337, 2.543)	-1%	28%
	Replicator 1 (unblinded)	1.943	(1.487, 2.537)	8%	6%
	Replicator 2 (unblinded)	1.943	(1.488, 2.537)	8%	6%
Ioannou 2020	Original (report)	-0.29	(-0.84, 0.25)	NA	NA
	Replicator 1 (blinded)	-0.319	(-1.058, 0.419)	10%	36%
	Replicator 2 (blinded)	-0.332	(-1.022, 0.358)	14%	27%
	Replicator 1 (unblinded)	-0.267	(-0.962, 0.428)	-8%	28%
	Replicator 2 (unblinded)	-0.276	(-0.961, 0.409)	-5%	26%
Yekeduz 2020	Original (report)	1.85	(1.26, 2.71)	NA	NA
	Replicator 1 (blinded)	1.533	(1.119, 2.10)	-31%	-18%
	Replicator 2 (blinded)	1.533	(1.118, 2.10)	-31%	-18%
	Replicator 1 (unblinded)	1.846	(1.26, 2.705)	0%	0%
	Replicator 2 (unblinded)	1.847	(1.26, 2.705)	0%	0%
Zhou 2021	Original (report)	2.81	(1.14, 4.48)	NA	NA
	Replicator 1 (blinded)	3.621	(2.311, 4.93)	29%	-22%
	Replicator 2 (blinded)	3.62	(2.326, 4.913)	29%	-23%
	Replicator 1 (unblinded)	3.381	(2.211, 4.551)	20%	-30%
	Replicator 2 (unblinded)	3.376	(2.224, 4.528)	20%	-31%
Minozzi 2020	Original (report)	0.66	(0.37, 1.2)	NA	NA
	Replicator 1 (blinded)	0.662	(0.366, 1.197)	-1%	1%
	Replicator 2 (blinded)	0.661	(0.367, 1.193)	0%	0%
	Replicator 1 (unblinded)	0.662	(0.366, 1.197)	-1%	1%
	Replicator 2 (unblinded)	0.661	(0.367, 1.193)	0%	0%
Wang 2020	Original (report)	1.05	(0.9, 1.21)	NA	NA
	Replicator 1 (blinded)	1.055	(0.944, 1.182)	10%	-24%
	Replicator 2 (blinded)	1.058	(0.946, 1.183)	16%	-24%
	Replicator 1 (unblinded)	1.069	(0.929, 1.23)	37%	-5%
	Replicator 2 (unblinded)	1.068	(0.929, 1.229)	35%	-5%
AlAnouti 2020	Original (report)	30.67	(4.89, 56.45)	NA	NA
	Replicator 1 (blinded)	0.368	(-9.185, 9.922)	-99%	-63%
	Replicator 2 (blinded)	0.46	(-9.048, 9.969)	-99%	-63%
	Replicator 1 (unblinded)	29.473	(4.236, 54.71)	-4%	-2%
	Replicator 2 (unblinded)	30.022	(4.937, 55.107)	-2%	-3%

Supplementary Table 5 (cont). Percentage differences between the original review's and replicators' summary estimate and confidence interval widths. Note: Ratio measures were log-transformed (natural log) prior to calculation of the percentage difference.

				Percentage difference	
**Review**	**Analysis**	**Summary estimate**	**95% CI**	**Summary estimate**	**CI width**
Goldberg 2020	Original (report)	1.08	(0.74, 1.43)	NA	NA
	Replicator 1 (blinded)	0.839	(0.308, 1.37)	-22%	54%
	Replicator 2 (blinded)	0.842	(0.31, 1.375)	-22%	54%
	Replicator 1 (unblinded)	0.839	(0.308, 1.37)	-22%	54%
	Replicator 2 (unblinded)	0.842	(0.31, 1.375)	-22%	54%
Jakubczyk 2020	Original (report)	2.7	(0.06, 5.34)	NA	NA
	Replicator 1 (blinded)	2.683	(0.082, 5.284)	-1%	-1%
	Replicator 2 (blinded)	2.696	(0.058, 5.335)	0%	0%
	Replicator 1 (unblinded)	2.683	(0.082, 5.284)	-1%	-1%
	Replicator 2 (unblinded)	2.696	(0.058, 5.335)	0%	0%
Gingold-Belfer 2020	Original (report)	0.89	(0.44, 1.79)	NA	NA
	Replicator 1 (blinded)	0.869	(0.43, 1.759)	20%	0%
	Replicator 2 (blinded)	0.87	(0.43, 1.759)	20%	0%
	Replicator 1 (unblinded)	0.869	(0.43, 1.759)	20%	0%
	Replicator 2 (unblinded)	0.87	(0.43, 1.759)	20%	0%

Supplementary Table 6. Discordance between P values for the test for overall effect from the original review's and replicators' meta-analyses.

**Review**	**Analysis**	**P value (overall)**	**P < 0.01**	**0.01 ≤ P < 0.05**	**0.05 ≤ P < 0.1**	**P ≥ 0.1**
Dohos 2020	Original (report)	0	Yes	-	-	-
	Replicator 1 (blinded)	0	Yes	-	-	-
	Replicator 2 (blinded)	0	Yes	-	-	-
	Replicator 1 (unblinded)	0	Yes	-	-	-
	Replicator 2 (unblinded)	0	Yes	-	-	-
Ioannou 2020	Original (report)	0.29	-	-	-	Yes
	Replicator 1 (blinded)	0.397	-	-	-	Yes
	Replicator 2 (blinded)	0.346	-	-	-	Yes
	Replicator 1 (unblinded)	0.452	-	-	-	Yes
	Replicator 2 (unblinded)	0.43	-	-	-	Yes
Yekeduz 2020	Original (report)	0	Yes	-	-	-
	Replicator 1 (blinded)	0.008	Yes	-	-	-
	Replicator 2 (blinded)	0.008	Yes	-	-	-
	Replicator 1 (unblinded)	0.002	Yes	-	-	-
	Replicator 2 (unblinded)	0.002	Yes	-	-	-
Zhou 2021	Original (report)	0	Yes	-	-	-
	Replicator 1 (blinded)	0	Yes	-	-	-
	Replicator 2 (blinded)	0	Yes	-	-	-
	Replicator 1 (unblinded)	0	Yes	-	-	-
	Replicator 2 (unblinded)	0	Yes	-	-	-
Minozzi 2020	Original (report)	0.17	-	-	-	Yes
	Replicator 1 (blinded)	0.172	-	-	-	Yes
	Replicator 2 (blinded)	0.169	-	-	-	Yes
	Replicator 1 (unblinded)	0.172	-	-	-	Yes
	Replicator 2 (unblinded)	0.169	-	-	-	Yes
Wang 2020	Original (report)	0.56	-	-	-	Yes
	Replicator 1 (blinded)	0.342	-	-	-	Yes
	Replicator 2 (blinded)	0.326	-	-	-	Yes
	Replicator 1 (unblinded)	0.352	-	-	-	Yes
	Replicator 2 (unblinded)	0.356	-	-	-	Yes
AlAnouti 2020	Original (report)	0.02	-	Yes	-	-
	Replicator 1 (blinded)	0.94	-	-	-	Yes
	Replicator 2 (blinded)	0.925	-	-	-	Yes
	Replicator 1 (unblinded)	0.022	-	Yes	-	-
	Replicator 2 (unblinded)	0.019	-	Yes	-	-

Supplementary Table 6 (cont). Discordance between P values for the test for overall effect from the original review's and replicators' meta-analyses.

Review	Analysis	P value (overall)	P < 0.01	0.01 ≤ P < 0.05	0.05 ≤ P < 0.1	P ≥ 0.1
Goldberg 2020	Original (estimated in R)	0	Yes	-	-	-
	Replicator 1 (blinded)	0.002	Yes	-	-	-
	Replicator 2 (blinded)	0.002	Yes	-	-	-
	Replicator 1 (unblinded)	0.002	Yes	-	-	-
	Replicator 2 (unblinded)	0.002	Yes	-	-	-
Jakubczyk 2020	Original (report)	0.045	-	Yes	-	-
	Replicator 1 (blinded)	0.043	-	Yes	-	-
	Replicator 2 (blinded)	0.045	-	Yes	-	-
	Replicator 1 (unblinded)	0.043	-	Yes	-	-
	Replicator 2 (unblinded)	0.045	-	Yes	-	-
Gingold-Belfer 2020	Original (report)	0.73	-	-	-	Yes
	Replicator 1 (blinded)	0.698	-	-	-	Yes
	Replicator 2 (blinded)	0.698	-	-	-	Yes
	Replicator 1 (unblinded)	0.698	-	-	-	Yes
	Replicator 2 (unblinded)	0.698	-	-	-	Yes

Supplementary Table 7. Factors associated with the eight blinded replicated meta-analyses classified as not fully replicable. (Note: '+' refers to suspected minor contributors to replication failure and '++' refers to suspected major contributors to replication failure.)

	Replicator-related		Error	Reporting			Other			
	Coordinator error	Replicator error	Reviewer error	Incomplete eligibility criteria	Ambiguous eligibility criteria	Incomplete data selection methods	Extraction of data from plots	Software-related differences	Unavailable data	Unknown reasons
AlAnouti 2020	++	-	+	-	-	-	-	-	-	-
Dohos 2020	-	++	-	-	-	+	-	-	-	-
Gingold-Belfer 2020	-	-	++	-	-	-	-	-	-	-
Goldberg 2020	-	-	-	-	-	++	-	-	-	-
Ioannou 2020	-	-	++	++	++	-	+	-	++	-
Wang 2020	++	-	-	-	-	-	++	+	-	-
Yekeduz 2020	-	++	-	-	-	-	-	+	-	-
Zhou 2021	-	-	-	-	++	-	-	+	-	++

Supplementary Table 8. Factors associated with the five unblinded replicated meta-analyses classified as not fully replicable. (Note: '+' refers to suspected minor contributors to replication failure and '++' refers to suspected major contributors to replication failure.)

	Replicator-related		Error	Reporting			Other			
	Coordinator error	Replicator error	Reviewer data extraction error	Incomplete eligibility criteria	Ambiguous eligibility criteria	Incomplete data selection methods	Extraction of data from plots	Software-related differences	Unavailable data	Unknown reasons
Gingold-Belfer 2020	-	-	++	-	-	-	-	-	-	-
Goldberg 2020	-	-	-	-	-	++	-	-	-	-
Ioannou 2020	-	-	++	-	++	-	+	-	-	-
Wang 2020	-	-	-	-	-	-	++	+	-	-
Zhou 2021	-	-	-	-	++	-	-	+	-	++

SUPPLEMENTARY FIGURES

Supplementary Figure 1. Scatter plot of the number of records retrieved by the original reviewers (square symbols) and the first (plus signs) and second (cross signs) replicators by database. Note: The y-axis is presented on a logarithmic scale (base 10).

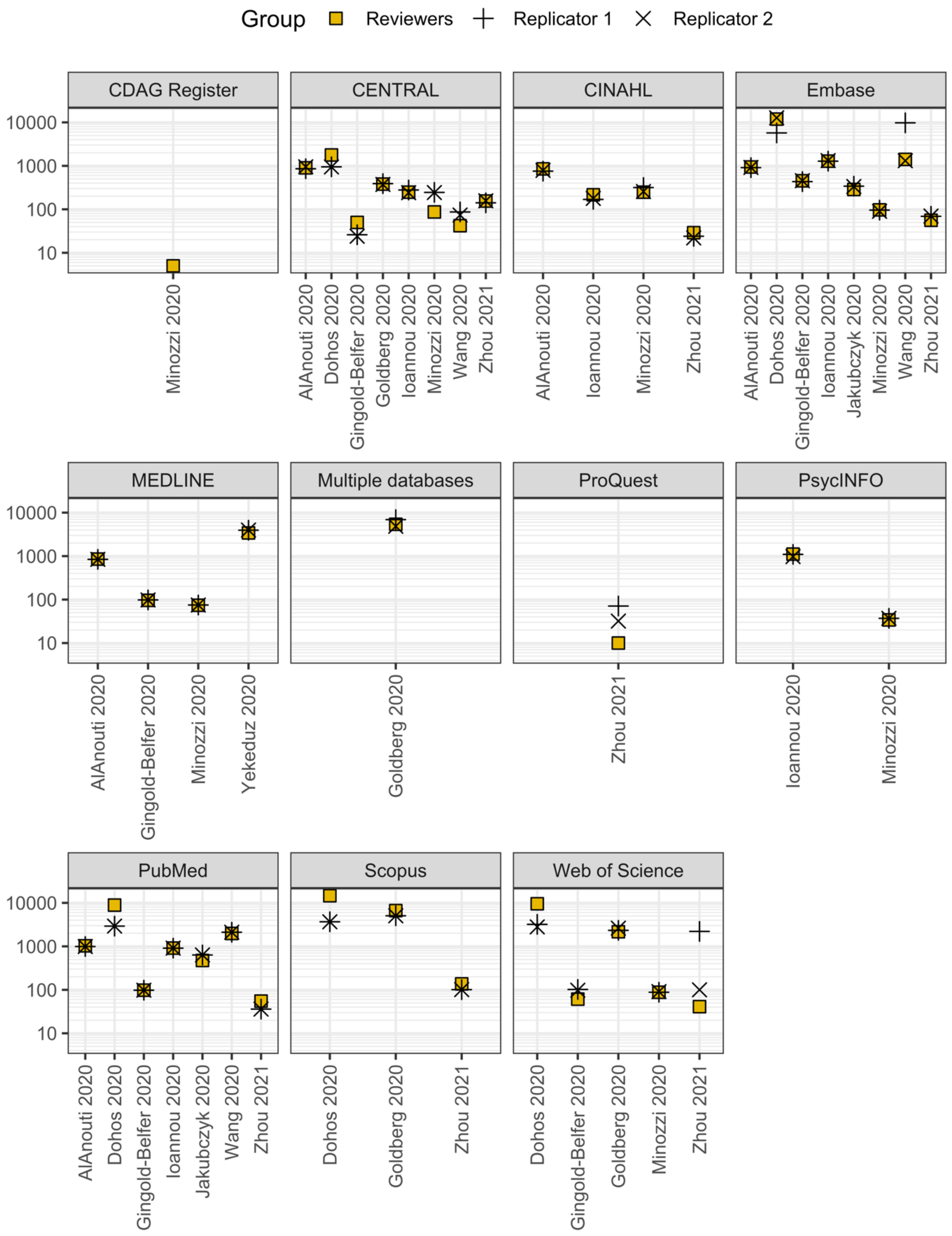

Supplementary Figure 2. Results of the original and replicated index meta-analyses for the review by AlAnouti et al. (2020). (Green shade: Met the criteria for 'fully replicable'. Red shade: Met the criteria for not 'fully replicable'. Black shade: Met the criteria for not 'fully replicable' and 'meaningfully' different.)

	Intervention			Control							
	Mean	SD	Total	Mean	SD	Total	MD	95% CI	Screening discrepancy	Data discrepancy	Reason for discrepancy
Original reviewers											
Farag et al. (2019)	233.8	97.0	24	158.6	35.4	25	75.20	(33.99, 116.41)	-	-	-
Wongwiwatthananukit (2013)	137.8	53.5	28	135.8	71.4	28	2.04	(-31.00, 35.08)	-	-	-
Yin et al. (2016)	NI	NI	NI	NI	NI	NI	NI	NI	-	-	-
[Fixed effect model]	-	-	-	-	-	-	30.67	(4.89, 56.45)			
Replicators (Blinded)											
Farag et al. (2019)	233.8	97.0	24	158.6	35.4	25	75.20	(33.99, 116.41)	No	No	-
Wongwiwatthananukit (2013)	137.8	53.5	30	135.8	71.4	30	2.04	(-29.88, 33.96)	No	Yes	Reviewer extraction error
Yin et al. (2016)	250.4	36.3	61	254.9	19.5	62	-4.43	(-14.74, 5.88)	Yes	-	Coordinator extraction error
[Fixed effect model (R1)]	-	-	-	-	-	-	0.37	(-9.19, 9.92)			
[Fixed effect model (R2)]	-	-	-	-	-	-	0.46	(-9.05, 9.97)			
Replicators (Unblinded)											
Farag et al. (2019)	233.8	97.0	24	158.6	35.4	25	75.20	(33.99, 116.41)	No	No	-
Wongwiwatthananukit (2013)	137.8	53.5	30	135.8	71.4	30	2.04	(-29.88, 33.96)	No	Yes	Reviewer extraction error
Yin et al. (2016)	NI	NI	NI	NI	NI	NI	NI	NI	No	-	-
[Fixed effect model (R1)]	-	-	-	-	-	-	29.47	(4.24, 54.71)			
[Fixed effect model (R2)]	-	-	-	-	-	-	30.02	(4.94, 55.11)			

Supplementary Figure 3. Results of the original and replicated index meta-analyses for the review by Dohos et al (2021). (Green shade: Met the criteria for 'fully replicable'. Red shade: Met the criteria for not 'fully replicable'. Black shade: Met the criteria for not 'fully replicable' and 'meaningfully' different.)

	Intervention		Control						
	Events	Total	Events	Total	RR	95% CI	Screening discrepancy	Data discrepancy	Reason for discrepancy
Original reviewers									
Feagan et al. (2000)	22	36	14	40	1.75	(1.06, 2.87)	-	-	-
Wenzl et al. (2014)	8	26	4	26	2.00	(0.69, 5.83)	-	-	-
Candy et al. (1995)	17	19	10	24	2.15	(1.31, 3.53)	-	-	-
O'Donoghue et al. (1978)	11	27	4	24	2.44	(0.90, 6.67)	-	-	-
Vilien et al. (2004)	8	15	3	14	2.49	(0.82, 7.55)	-	-	-
Lémann et al. (2005)	9	43	3	40	2.79	(0.81, 9.58)	-	-	-
Hawthorne et al. (1992)	20	34	14	33	1.39	(0.85, 2.26)	-	-	-
[Random effects model]	-	-	-	-	1.85	(1.44, 2.38)			
Replicators (Blinded)									
Feagan et al. (2000)	22	36	14	40	1.75	(1.06, 2.87)	No	No	-
Wenzl et al. (2014)	8	26	4	26	2.00	(0.69, 5.83)	No	No	-
Candy et al. (1995)	NI	NI	NI	NI	NI	NI	Yes	-	Replicator screening error
O'Donoghue et al. (1978)	NI	NI	NI	NI	NI	NI	Yes	-	Replicator screening error
Vilien et al. (2004)	8	15	2	13	3.47	(0.89, 13.51)	No	Yes	Incomplete data selection methods
Lémann et al. (2005)	9	43	3	40	2.79	(0.81, 9.58)	No	No	-
Hawthorne et al. (1992)	20	34	12	33	1.62	(0.95, 2.75)	No	Yes	Incomplete data selection methods
[Random effects model (R1)]	-	-	-	-	1.84	(1.34, 2.55)			
[Random effects model (R2)]	-	-	-	-	1.84	(1.34, 2.54)			
Replicators (Unblinded)									
Feagan et al. (2000)	22	36	14	40	1.75	(1.06, 2.87)	No	No	-
Wenzl et al. (2014)	8	26	4	26	2.00	(0.69, 5.83)	No	No	-
Candy et al. (1995)	17	19	10	24	2.15	(1.31, 3.53)	No	No	-
O'Donoghue et al. (1978)	9	25	1	21	7.56	(1.04, 54.91)	No	Yes	Incomplete data selection methods
Vilien et al. (2004)	8	15	2	13	3.47	(0.89, 13.51)	No	Yes	Incomplete data selection methods
Lémann et al. (2005)	9	43	3	40	2.79	(0.81, 9.58)	No	No	-
Hawthorne et al. (1992)	20	34	12	31	1.52	(0.90, 2.57)	No	Yes	Incomplete data selection methods
[Random effects model (R1)]	-	-	-	-	1.94	(1.49, 2.54)			
[Random effects model (R2)]	-	-	-	-	1.94	(1.49, 2.54)			

Supplementary Figure 4. Results of the original and replicated index meta-analyses for the review by Gingold-Belfer et al. (2021). (Green shade: Met the criteria for 'fully replicable'. Red shade: Met the criteria for not 'fully replicable'. Black shade: Met the criteria for not 'fully replicable' and 'meaningfully' different.)

	Intervention		Control				Screening discrepancy	Data discrepancy	Reason for discrepancy
	Events	Total	Events	Total	OR	95% CI			
Original reviewers									
Hung et al. (2015)	67	76	68	75	0.77	(0.27, 2.18)	-	-	-
Miehlke et al. (2006)	54	73	50	72	1.25	(0.61, 2.58)	-	-	-
Navarro-Jarabo et al. (2007)	20	45	38	54	0.34	(0.15, 0.77)	-	-	-
Perri et al. (2001)	69	90	60	90	1.64	(0.85, 3.17)	-	-	-
[Random effects model]	-	-	-	-	0.89	(0.44, 1.79)			
Replicators (Blinded)									
Hung et al. (2015)	67	76	68	75	0.77	(0.27, 2.18)	No	No	-
Miehlke et al. (2006)	54	73	50	72	1.25	(0.61, 2.58)	No	No	-
Navarro-Jarabo et al. (2007)	20	45	38	54	0.34	(0.15, 0.77)	No	No	-
Perri et al. (2001)	69	90	30	45	1.64	(0.75, 3.62)	No	Yes	Reviewer extraction error
[Random effects model (R1)]	-	-	-	-	0.87	(0.43, 1.76)			
[Random effects model (R2)]	-	-	-	-	0.87	(0.43, 1.76)			
Replicators (Unblinded)									
Hung et al. (2015)	67	76	68	75	0.77	(0.27, 2.18)	No	No	-
Miehlke et al. (2006)	54	73	50	72	1.25	(0.61, 2.58)	No	No	-
Navarro-Jarabo et al. (2007)	20	45	38	54	0.34	(0.15, 0.77)	No	No	-
Perri et al. (2001)	69	90	30	45	1.64	(0.75, 3.62)	No	Yes	Reviewer extraction error
[Random effects model (R1)]	-	-	-	-	0.87	(0.43, 1.76)			
[Random effects model (R2)]	-	-	-	-	0.87	(0.43, 1.76)			

Supplementary Figure 5. Results of the original and replicated index meta-analyses for the review by Goldberg et al. (2020). (Green shade: Met the criteria for 'fully replicable'. Red shade: Met the criteria for not 'fully replicable'. Black shade: Met the criteria for not 'fully replicable' and 'meaningfully' different.)

	Intervention			Control							
	Mean	SD	Total	Mean	SD	Total	Cohen's d	95% CI	Screening discrepancy	Data discrepancy	Reason for discrepancy
Original reviewers											
Gasser et al. (2014)	-	-	-	-	-	-	2.22	(0.91, 3.53)*	-	-	-
Griffiths et al. (2016)	-	-	-	-	-	-	0.86	(0.24, 1.48)*	-	-	-
Grob et al. (2011)	-	-	-	-	-	-	0.94	(-0.06, 1.94)*	-	-	-
Palhano-Fontes et al. (2019)	-	-	-	-	-	-	1.20	(0.52, 1.88)*	-	-	-
Ross et al. (2016)	-	-	-	-	-	-	0.98	(0.27, 1.69)*	-	-	-
[Random effects model]	-	-	-	-	-	-	1.08	(0.74, 1.43)			
Replicators (Blinded)											
Gasser et al. (2014)	6.6	37.7	8	3.4	24.3	3	0.09	(-1.24, 1.42)	No	Yes	Incomplete data selection methods
Griffiths et al. (2016)	11.6	5.8	26	8.5	5.4	25	0.55	(-0.01, 1.11)	No	Yes	Incomplete data selection methods
Grob et al. (2011)	9.7	7.8	6	0.1	8.4	6	1.21	(-0.04, 2.46)	No	Yes	Incomplete data selection methods
Palhano-Fontes et al. (2019)	14.4	6.6	14	2.8	6.4	15	1.77	(0.90, 2.64)	No	Yes	Incomplete data selection methods
Ross et al. (2016)	11.7	10.3	14	5.5	10.1	15	0.61	(-0.14, 1.35)	No	Yes	Incomplete data selection methods
[Random effects model (R1)]	-	-	-	-	-	-	0.84	(0.31, 1.37)			
[Random effects model (R2)]	-	-	-	-	-	-	0.84	(0.31, 1.38)			
Replicators (Unblinded)											
Gasser et al. (2014)	6.6	37.7	8	3.4	24.3	3	0.09	(-1.24, 1.42)	No	Yes	Incomplete data selection methods
Griffiths et al. (2016)	11.6	5.8	26	8.5	5.4	25	0.55	(-0.01, 1.11)	No	Yes	Incomplete data selection methods
Grob et al. (2011)	9.7	7.8	6	0.1	8.4	6	1.21	(-0.04, 2.46)	No	Yes	Incomplete data selection methods
Palhano-Fontes et al. (2019)	14.4	6.6	14	2.8	6.4	15	1.77	(0.90, 2.64)	No	Yes	Incomplete data selection methods
Ross et al. (2016)	11.7	10.3	14	5.5	10.1	15	0.61	(-0.14, 1.35)	No	Yes	Incomplete data selection methods
[Random effects model (R1)]	-	-	-	-	-	-	0.84	(0.31, 1.37)			
[Random effects model (R2)]	-	-	-	-	-	-	0.84	(0.31, 1.38)			

Supplementary Figure 6. Results of the original and replicated index meta-analyses for the review by Ioannou et al. (2021). (Green shade: Met the criteria for 'fully replicable'. Red shade: Met the criteria for not 'fully replicable'. Black shade: Met the criteria for not 'fully replicable' and 'meaningfully' different.)

	Intervention			Control					Screening discrepancy	Data discrepancy	Reason for discrepancy
	Mean	SD	Total	Mean	SD	Total	Hedge's g	95% CI			
Original reviewers											
Benedetti et al. (1997)	9.6	11.4	5	16.4	12.9	5	-0.51	(-1.78, 0.77)	-	-	-
Elsenga et al. (1982)	21.5	1.6	10	23.2	1.6	10	-1.03	(-1.98, -0.09)	-	-	-
Kragh et al. (2017a)	17.4	5.2	32	20.2	5.5	32	-0.52	(-1.02, -0.02)	-	-	-
Kundermann et al. (2008)	16.3	6.6	9	17.8	9.4	10	-0.17	(-1.07, 0.73)	-	-	-
Reynolds et al. (2005)	15.1	3.9	27	12.1	4.1	26	0.74	(0.18, 1.29)	-	-	-
Wu et al. (2009)	10.2	7.3	32	14.4	8.0	17	-0.55	(-1.15, 0.05)	-	-	-
[Random effects model]	-	-	-	-	-	-	-0.29	(-0.84, 0.25)			
Replicators (Blinded)											
Benedetti et al. (1997)	14.1	10.7	5	22.1	12.7	5	-0.61	(-1.90, 0.67)	No	Yes	Reviewer extraction error
Elsenga et al. (1982)	NI	NI	NI	NI	NI	NI	NI	NI	Yes	-	Incomplete eligibility criteria
Kragh et al. (2017a)	NI	NI	NI	NI	NI	NI	NI	NI	Yes	-	Unclear eligibility criteria
Kundermann et al. (2008)	16.3	6.6	9	17.8	9.5	10	-0.17	(-1.08, 0.73)	No	Yes	Data extraction from plot
Reynolds et al. (2005)	NI	NI	NI	NI	NI	NI	NI	NI	Yes	-	Unable to obtain data
Wu et al. (2009)	NI	NI	NI	NI	NI	NI	NI	NI	Yes	-	Incomplete eligibility criteria
[Random effects model (R1)]	-	-	-	-	-	-	-0.32	(-1.06, 0.42)			
[Random effects model (R2)]	-	-	-	-	-	-	-0.33	(-1.02, 0.36)			
Replicators (Unblinded)											
Benedetti et al. (1997)	14.1	10.7	5	22.1	12.7	5	-0.61	(-1.90, 0.67)	No	Yes	Reviewer extraction error
Elsenga et al. (1982)	21.5	1.6	10	23.2	1.6	10	-1.03	(-1.98, -0.09)	No	No	-
Kragh et al. (2017a)	NI	NI	NI	NI	NI	NI	NI	NI	Yes	-	Unclear eligibility criteria
Kundermann et al. (2008)	16.3	6.6	9	17.8	9.5	10	-0.17	(-1.08, 0.73)	No	Yes	Data extraction from plot
Reynolds et al. (2005)	15.1	3.9	27	12.1	4.1	26	0.74	(0.18, 1.29)	No	No	-
Wu et al. (2009)	10.2	7.3	32	14.4	8.0	17	-0.55	(-1.15, 0.05)	No	No	-
[Random effects model (R1)]	-	-	-	-	-	-	-0.27	(-0.96, 0.43)			
[Random effects model (R2)]	-	-	-	-	-	-	-0.28	(-0.96, 0.41)			

Supplementary Figure 7. Results of the original and replicated index meta-analyses for the review by Jakubczuk et al. (2020). (Green shade: Met the criteria for 'fully replicable'. Red shade: Met the criteria for not 'fully replicable'. Black shade: Met the criteria for not 'fully replicable' and 'meaningfully' different.)

	Intervention			Control							
	Mean	SD	Total	Mean	SD	Total	Cohen's d	95% CI	Screening discrepancy	Data discrepancy	Reason for discrepancy
Original reviewers											
Alizadeh et al. (2017)	-	-	-	-	-	-	8.24	(6.63, 9.86)	-	-	-
Nasseri et al. (2017)	-	-	-	-	-	-	0.14	(-0.36, 0.65)	-	-	-
Saraf-Bank et al. (2019)	-	-	-	-	-	-	0.28	(-0.23, 0.79)	-	-	-
[Random effects model]	-	-	-	-	-	-	2.70	(0.06, 5.34)			
Replicators (Blinded)											
Gasser et al. (2014)	2.0	0.1	28	1.4	0.1	28	8.24	(6.63, 9.86)	No	No	-
Griffiths et al. (2016)	208.0	91.7	31	196.1	72.6	30	0.14	(-0.36, 0.65)	No	No	-
Grob et al. (2011)	230.0	40.0	30	220.0	30.0	30	0.28	(-0.23, 0.79)	No	No	-
[Random effects model (R1)]	-	-	-	-	-	-	2.68	(0.08, 5.28)			
[Random effects model (R2)]	-	-	-	-	-	-	2.70	(0.06, 5.34)			
Replicators (Unblinded)											
Gasser et al. (2014)	2.0	0.1	28	1.4	0.1	28	8.24	(6.63, 9.86)	No	No	-
Griffiths et al. (2016)	208.0	91.7	31	196.1	72.6	30	0.14	(-0.36, 0.65)	No	No	-
Grob et al. (2011)	230.0	40.0	30	220.0	30.0	30	0.28	(-0.23, 0.79)	No	No	-
[Random effects model (R1)]	-	-	-	-	-	-	2.68	(0.08, 5.28)			
[Random effects model (R2)]	-	-	-	-	-	-	2.70	(0.06, 5.34)			

Supplementary Figure 8. Results of the original and replicated index meta-analyses for the review by Minozzi et al. (2020). (Green shade: Met the criteria for 'fully replicable'. Red shade: Met the criteria for not 'fully replicable'. Black shade: Met the criteria for not 'fully replicable' and 'meaningfully' different.)

	Intervention		Control				Screening discrepancy	Data discrepancy	Reason for discrepancy
	Events	Total	Events	Total	RR	95% CI			
Original reviewers									
Fischer et al. (2006)	3	9	1	9	3.00	(0.38, 23.68)	-	-	-
Jones et al. (2005)	4	15	6	15	0.67	(0.23, 1.89)	-	-	-
Jones et al. (2010)	16	89	28	86	0.55	(0.32, 0.95)	-	-	-
[Random effects model]	-	-	-	-	0.66	(0.37, 1.20)			
Replicators (Blinded)									
Fischer et al. (2006)	3	9	1	9	3.00	(0.38, 23.68)	No	No	-
Jones et al. (2005)	4	15	6	15	0.67	(0.23, 1.89)	No	No	-
Jones et al. (2010)	16	89	28	86	0.55	(0.32, 0.95)	No	No	-
[Random effects model (R1)]	-	-	-	-	0.66	(0.37, 1.20)			
[Random effects model (R2)]	-	-	-	-	0.66	(0.37, 1.19)			
Replicators (Unblinded)									
Fischer et al. (2006)	3	9	1	9	3.00	(0.38, 23.68)	No	No	-
Jones et al. (2005)	4	15	6	15	0.67	(0.23, 1.89)	No	No	-
Jones et al. (2010)	16	89	28	86	0.55	(0.32, 0.95)	No	No	-
[Random effects model (R1)]	-	-	-	-	0.66	(0.37, 1.20)			
[Random effects model (R2)]	-	-	-	-	0.66	(0.37, 1.19)			

Supplementary Figure 9. Results of the original and replicated index meta-analyses for the review by Wang et al. (2020). (Green shade: Met the criteria for 'fully replicable'. Red shade: Met the criteria for not 'fully replicable'. Black shade: Met the criteria for not 'fully replicable' and 'meaningfully' different.)

	HR	95% CI	Screening discrepancy	Data discrepancy	Reason for discrepancy
Original reviewers					
Akiyama et al. (1994)	NI	NI	-	-	-
Fan et al. (2019)	1.02	(0.66, 1.57)	-	-	-
Fujita et al. (1995)	1.13	(0.67, 1.92)	-	-	-
Fujita et al. (2003)	NI	NI	-	-	-
Igaki et al. (2004)	1.29	(0.77, 2.15)	-	-	-
Koterazawa et al. (2019)	NI	NI	-	-	-
Li et al. (2012)	0.88	(0.63, 1.23)	-	-	-
Shao et al. (2018)	0.94	(0.71, 1.24)	-	-	-
Shim et al. (2010)	1.35	(0.71, 2.58)	-	-	-
Tabira et al. (1999)	1.20	(0.65, 2.20)	-	-	-
Zhang et al. (2008)	1.31	(0.82, 2.10)	-	-	-
[Fixed effect model]	1.05	(0.90, 1.21)			
Replicators (Blinded)					
Akiyama et al. (1994)	1.54	(1.22, 1.95)	Yes	-	Coordinator extraction error
Fan et al. (2019)	1.01	(0.63, 1.62)	No	Trivial	Software-related
Fujita et al. (1995)	1.19	(0.77, 1.83)	No	Yes	Data extraction from plot
Fujita et al. (2003)	0.36	(0.25, 0.53)	Yes	-	Coordinator extraction error
Igaki et al. (2004)	1.25	(0.80, 1.96)	No	Yes	Data extraction from plot
Koterazawa et al. (2019)	1.15	(0.68, 1.95)	Yes	-	Coordinator extraction error
Li et al. (2012)	0.88	(0.62, 1.26)	No	Yes	Data extraction from plot
Shao et al. (2018)	0.93	(0.72, 1.19)	No	Trivial	Software-related
Shim et al. (2010)	1.15	(0.64, 2.07)	No	Yes	Data extraction from plot
Tabira et al. (1999)	1.52	(0.89, 2.57)	No	Yes	Data extraction from plot
Zhang et al. (2008)	1.30	(0.86, 1.96)	No	Yes	Data extraction from plot
[Fixed effect model (R1)]	1.06	(0.94, 1.18)			
[Fixed effect model (R2)]	1.06	(0.95, 1.18)			
Replicators (Unblinded)					
Akiyama et al. (1994)	NI	NI	No	-	-
Fan et al. (2019)	1.01	(0.63, 1.62)	No	Trivial	Software-related
Fujita et al. (1995)	1.19	(0.77, 1.83)	No	Yes	Data extraction from plot
Fujita et al. (2003)	NI	NI	No	-	-
Igaki et al. (2004)	1.25	(0.80, 1.96)	No	Yes	Data extraction from plot
Koterazawa et al. (2019)	NI	NI	No	-	-
Li et al. (2012)	0.88	(0.62, 1.26)	No	Yes	Data extraction from plot
Shao et al. (2018)	0.93	(0.72, 1.19)	No	Trivial	Software-related
Shim et al. (2010)	1.15	(0.64, 2.07)	No	Yes	Data extraction from plot
Tabira et al. (1999)	1.52	(0.89, 2.57)	No	Yes	Data extraction from plot
Zhang et al. (2008)	1.30	(0.86, 1.96)	No	Yes	Data extraction from plot
[Fixed effect model (R1)]	1.07	(0.93, 1.23)			
[Fixed effect model (R2)]	1.07	(0.93, 1.23)			

Supplementary Figure 10. Results of the original and replicated index meta-analyses for the review by Yekeduz et al. (2020). (Green shade: Met the criteria for ‘fully replicable’. Red shade: Met the criteria for not ‘fully replicable’. Black shade: Met the criteria for not ‘fully replicable’ and ‘meaningfully’ different.)

	aOR	95% CI	Screening discrepancy	Data discrepancy	Reason for discrepancy
Original reviewers					
Kuderer et al. (2000)	1.47	(0.84, 2.57)	-	-	-
Lee et al. (2020a)	2.09	(1.09, 4.01)	-	-	-
Lee et al. (2020b)	NI	NI	-	-	-
Yang et al. (2020)	3.51	(1.16, 10.62)	-	-	-
Yarza et al. (2020)	1.60	(0.40, 6.40)	-	-	-
[Random effects model]	1.85	(1.26, 2.71)			
Replicators (Blinded)					
Kuderer et al. (2000)	1.47	(0.84, 2.56)	No	Trivial	Software-related
Lee et al. (2020a)	2.09	(1.09, 4.08)	No	Trivial	Software-related
Lee et al. (2020b)	1.18	(0.81, 1.72)	Yes	-	Replicator screening error
Yang et al. (2020)	3.51	(1.16, 10.59)	No	Trivial	Software-related
Yarza et al. (2020)	1.60	(0.40, 6.33)	No	Trivial	Software-related
[Random effects model (R1)]	1.53	(1.12, 2.10)			
[Random effects model (R2)]	1.53	(1.12, 2.10)			
Replicators (Unblinded)					
Kuderer et al. (2000)	1.47	(0.84, 2.56)	No	Trivial	Software-related
Lee et al. (2020a)	2.09	(1.09, 4.08)	No	Trivial	Software-related
Lee et al. (2020b)	NI	NI	No	-	-
Yang et al. (2020)	3.51	(1.16, 10.59)	No	Trivial	Software-related
Yarza et al. (2020)	1.60	(0.40, 6.33)	No	Trivial	Software-related
[Random effects model (R1)]	1.85	(1.26, 2.71)			
[Random effects model (R2)]	1.85	(1.26, 2.71)			

Supplementary Figure 11. Results of the original and replicated index meta-analyses for the review by Zhou et al. (2021). (Green shade: Met the criteria for 'fully replicable'. Red shade: Met the criteria for not 'fully replicable'. Black shade: Met the criteria for not 'fully replicable' and 'meaningfully' different.) *Confidence intervals from the original review were estimated in R.

	Intervention			Control							
	Mean	SD	Total	Mean	SD	Total	Hedge's g	95% CI	Screening discrepancy	Data discrepancy	Reason for discrepancy
Original reviewers											
Hoseini et al. (2016)	NI	NI	NI	NI	NI	NI	NI	NI	-	-	-
Kajagar et al. (2012)	-	-	34	-	-	34	5.21	(4.22, 6.20)*	-	-	-
Kaviani et al. (2011)	-	-	13	-	-	10	2.00	(1.02, 2.98)*	-	-	-
Mathur et al. (2017)	-	-	15	-	-	15	2.98	(1.95, 4.01)*	-	-	-
Minatel et al. (2009)	-	-	7	-	-	7	2.29	(0.99, 3.59)*	-	-	-
Naidu et al. (2005)	-	-	8	-	-	8	4.17	(2.45, 5.89)*	-	-	-
Zhang et al. (2013)	-	-	42	-	-	42	0.45	(0.02, 0.88)*	-	-	-
[Random effects model]	-	-	-	-	-	-	2.81	(1.14, 4.48)*			
Replicators (Blinded)											
Hoseini et al. (2016)	44.0	6.4	15	6.5	8.0	12	5.11	(3.46, 6.76)	Yes	-	Unclear
Kajagar et al. (2012)	40.2	6.3	34	11.9	4.3	34	5.21	(4.19, 6.23)	No	Trivial	Software-related
Kaviani et al. (2011)	47.5	9.0	13	29.4	7.6	10	2.07	(1.02, 3.12)	No	Trivial	Software-related
Mathur et al. (2017)	37.3	8.8	15	15.2	5.4	15	2.94	(1.87, 4.01)	No	Trivial	Software-related
Minatel et al. (2009)	NI	NI	NI	NI	NI	NI	NI	NI	Yes	-	Ambiguous eligiblity criteria
Naidu et al. (2005)	41.3	15.7	8	4.6	6.0	8	2.92	(1.40, 4.43)	No	Yes	Unclear
Zhang et al. (2013)	NI	NI	NI	NI	NI	NI	NI	NI	Yes	-	Unclear eligiblity criteria
[Random effects model (R1)]							3.62	(2.31, 4.93)			
[Random effects model (R2)]							3.62	(2.33, 4.91)			
Replicators (Unblinded)											
Hoseini et al. (2016)	44.0	6.4	15	6.5	8.0	12	5.11	(3.46, 6.76)	Yes	-	Unclear
Kajagar et al. (2012)	40.2	6.3	34	11.9	4.3	34	5.21	(4.19, 6.23)	No	Trivial	Software-related
Kaviani et al. (2011)	47.5	9.0	13	29.4	7.6	10	2.07	(1.02, 3.12)	No	Trivial	Software-related
Mathur et al. (2017)	37.3	8.8	15	15.2	5.4	15	2.94	(1.87, 4.01)	No	Trivial	Software-related
Minatel et al. (2009)	44.1	21.6	7	-8.9	24.7	7	2.14	(0.74, 3.53)	No	Trivial	Software-related
Naidu et al. (2005)	41.3	15.7	8	4.6	6.0	8	2.92	(1.40, 4.43)	No	Yes	Unclear
Zhang et al. (2013)	NI	NI	NI	NI	NI	NI	NI	NI	Yes	-	Unclear eligiblity criteria
[Random effects model (R1)]							3.38	(2.21, 4.55)			
[Random effects model (R2)]							3.38	(2.22, 4.53)			

Supplementary Figure 12. Results of the original and sensitivity meta-analyses for the review by AlAnouti et al. (2020). (Green shade: Met the criteria for 'fully replicable'. Red shade: Met the criteria for not 'fully replicable'. Black shade: Met the criteria for not 'fully replicable' and 'meaningfully' different.)

	Intervention			Control					Screening discrepancy	Data discrepancy	Reason for discrepancy
	Mean	SD	Total	Mean	SD	Total	MD	95% CI			
Original reviewers											
Farag et al. (2019)	233.8	97.0	24	158.6	35.4	25	75.20	(33.99, 116.41)	-	-	-
Makariou et al. (2017)	NA	NA	NA	NA	NA	NA	NA	NA	-	-	-
Wongwiwatthananukit (2013)	137.8	53.5	28	135.8	71.4	28	2.04	(-31.00, 35.08)	-	-	-
Yin et al. (2016)	NI	NI	NI	NI	NI	NI	NI	NI	-	-	-
[Fixed effect model]	-	-	-	-	-	-	30.67	(4.89, 56.45)			
Replicators (Blinded)											
Farag et al. (2019)	233.8	97.0	24	158.6	35.4	25	75.20	(33.99, 116.41)	No	No	-
Makariou et al. (2017)	139.3	52.9	25	143.7	53.3	25	-4.40	(-33.84, 25.04)	NA	NA	NA
Wongwiwatthananukit (2013)	137.8	53.5	30	135.8	71.4	30	2.04	(-29.88, 33.96)	No	Yes	Reviewer extraction error
Yin et al. (2016)	250.4	36.3	61	254.9	19.5	62	-4.43	(-14.74, 5.88)	Yes	-	Coordinator extraction error
[Fixed effect model (R1)]	-	-	-	-	-	-	-0.09	(-9.17, 9.00)			
Replicators (Unblinded)											
Farag et al. (2019)	233.8	97.0	24	158.6	35.4	25	75.20	(33.99, 116.41)	No	No	-
Makariou et al. (2017)	139.3	52.9	25	143.7	53.3	25	-4.40	(-33.84, 25.04)	NA	NA	NA
Wongwiwatthananukit (2013)	137.8	53.5	30	135.8	71.4	30	2.04	(-29.88, 33.96)	No	Yes	Reviewer extraction error
Yin et al. (2016)	NI	NI	NI	NI	NI	NI	NI	NI	No	-	-
[Fixed effect model (R1)]	-	-	-	-	-	-	15.12	(-4.04, 34.28)			

Supplementary Figure 13. Results of the original and sensitivity meta-analyses for the review by Wang et al. (2020). (Green shade: Met the criteria for 'fully replicable'. Red shade: Met the criteria for not 'fully replicable'. Black shade: Met the criteria for not 'fully replicable' and 'meaningfully' different.)

	HR	95% CI	Screening discrepancy	Data discrepancy	Reason for discrepancy
Original reviewers					
Akiyama et al. (1994)	NI	NI	-	-	-
Fan et al. (2019)	1.02	(0.66, 1.57)	-	-	-
Fujita et al. (1995)	1.13	(0.67, 1.92)	-	-	-
Fujita et al. (2003)	NI	NI	-	-	-
Igaki et al. (2004)	1.29	(0.77, 2.15)	-	-	-
Koterazawa et al. (2019)	NI	NI	-	-	-
Li et al. (2012)	0.88	(0.63, 1.23)	-	-	-
Shao et al. (2018)	0.94	(0.71, 1.24)	-	-	-
Shim et al. (2010)	1.35	(0.71, 2.58)	-	-	-
Tabira et al. (1999)	1.20	(0.65, 2.20)	-	-	-
Zhang et al. (2008)	1.31	(0.82, 2.10)	-	-	-
[Fixed effect model]	1.05	(0.90, 1.21)			
Replicators (Blinded)					
Akiyama et al. (1994)	Exc	Exc	NA	NA	NA
Fan et al. (2019)	1.01	(0.63, 1.62)	No	Trivial	Software-related
Fujita et al. (1995)	Exc	Exc	NA	NA	NA
Fujita et al. (2003)	0.36	(0.25, 0.53)	Yes	-	Coordinator extraction error
Igaki et al. (2004)	Exc	Exc	NA	NA	NA
Koterazawa et al. (2019)	1.15	(0.68, 1.95)	Yes	-	Coordinator extraction error
Li et al. (2012)	0.88	(0.62, 1.26)	No	Yes	Data extraction from plot
Shao et al. (2018)	0.93	(0.72, 1.19)	No	Trivial	Software-related
Shim et al. (2010)	1.15	(0.64, 2.07)	No	Yes	Data extraction from plot
Tabira et al. (1999)	1.52	(0.89, 2.57)	No	Yes	Data extraction from plot
Zhang et al. (2008)	Exc	Exc	NA	NA	NA
[Fixed effect model (R1)]	1.01	(0.86, 1.19)			
Replicators (Unblinded)					
Akiyama et al. (1994)	NI	NI	No	-	-
Fan et al. (2019)	1.01	(0.63, 1.62)	No	Trivial	Software-related
Fujita et al. (1995)	Exc	Exc	NA	NA	NA
Fujita et al. (2003)	NI	NI	No	-	-
Igaki et al. (2004)	Exc	Exc	NA	NA	NA
Koterazawa et al. (2019)	NI	NI	No	-	-
Li et al. (2012)	0.88	(0.62, 1.26)	No	Yes	Data extraction from plot
Shao et al. (2018)	0.93	(0.72, 1.19)	No	Trivial	Software-related
Shim et al. (2010)	1.15	(0.64, 2.07)	No	Yes	Data extraction from plot
Tabira et al. (1999)	1.52	(0.89, 2.57)	No	Yes	Data extraction from plot
Zhang et al. (2008)	Exc	Exc	NA	NA	NA
[Fixed effect model (R1)]	0.99	(0.84, 1.18)			
